# Peptidoglycan: a post-genomic analysis

**DOI:** 10.1186/1471-2180-12-294

**Published:** 2012-12-18

**Authors:** Caroline Cayrou, Bernard Henrissat, Philippe Gouret, Pierre Pontarotti, Michel Drancourt

**Affiliations:** 1 Unité de Recherche sur les Maladies Infectieuses et Tropicales Emergentes, UMR CNRS 7872 IRD 198, Méditerranée Infection, Aix-Marseille-Université, Marseille, France; 2 Architecture et Fonction des Macromolécules Biologiques, Aix-Marseille Université, CNRS UMR 7257, Marseille, France; 3 Evolution Biologique et Modélisation, UMR-CNRS 6632, Université de Provence, Marseille, France

**Keywords:** Peptidoglycan, Genome, Glycoside hydrolase, Glycosyltransferase, Gram, Beta-lactamines, Glycopeptides

## Abstract

**Background:**

To derive post-genomic, neutral insight into the peptidoglycan (PG) distribution among organisms, we mined 1,644 genomes listed in the Carbohydrate-Active Enzymes database for the presence of a minimal 3-gene set that is necessary for PG metabolism. This gene set consists of one gene from the glycosyltransferase family GT28, one from family GT51 and at least one gene belonging to one of five glycoside hydrolase families (GH23, GH73, GH102, GH103 and GH104).

**Results:**

None of the 103 Viruses or 101 Archaea examined possessed the minimal 3-gene set, but this set was detected in 1/42 of the Eukarya members (*Micromonas* sp., coding for GT28, GT51 and GH103) and in 1,260/1,398 (90.1%) of Bacteria*,* with a 100% positive predictive value for the presence of PG. Pearson correlation test showed that GT51 family genes were significantly associated with PG with a value of 0.963 and a p value less than 10^-3^. This result was confirmed by a phylogenetic comparative analysis showing that the GT51-encoding gene was significantly associated with PG with a Pagel’s score of 60 and 51 (percentage of error close to 0%). Phylogenetic analysis indicated that the GT51 gene history comprised eight loss and one gain events, and suggested a dynamic on-going process.

**Conclusions:**

Genome analysis is a neutral approach to explore prospectively the presence of PG in uncultured, sequenced organisms with high predictive values.

## Background

The macromolecule peptidoglycan (PG) is a component of the bacterial cell wall that participates in withstanding osmotic pressure, maintaining the cell shape and anchoring other cell envelope components [1] PG is composed of linear glycan strands cross-linked by short peptides, with glycan strands of alternating N-acetylglucosamine (GlcNAc) and N-acetylmuramic acid (MurNAc) residues linked by β-1→4 bonds [1]. PG is at the basis of the first classification of bacteria using the staining procedure developed by Hans Christian Joachim Gram in 1884 [2]. This method reveals the presence of PG, with blue-colored Gram-positive bacteria having a thick PG layer, red-colored Gram-negative bacteria having a thin PG layer and poorly stained bacteria lacking PG. However, Gram staining lacks sensitivity and specificity for the detection of PG: for example, *Mycobacterium* organisms show variable results with Gram staining, despite the fact that they do have PG [3]. In addition, PG-less *Planctomycetes* and *Chlamydia* bacteria stain red like Gram-negative bacteria [4,5]*.* Further exploration of PG using electron microscopy observation of the cell wall refined previous optic microscopy observations, and biochemical analyses further allowed analyzing the cell wall PG composition, contributing to the description of additional Gram-positive species [6].

PG biosynthesis is a dynamic complex process involving 20 enzymatic reactions, including the formation of GlcNAc-MurNAc dimers by a glycosyltransferase (GT) of family GT28 (in this report, we adopted the family classification described in the CAZy database [7,8]) and the polymerization of the dimers to form the linear glycan strands by family GT51 glycosyltransferase [9]. These two glycosyltransferase families were the only ones evolved in the PG synthesis. Furthermore, PG lysis involves enzymes that may belong to six different glycoside hydrolase (GH) families, GH23, GH25, GH73, GH102, GH103 and GH104. Indeed, GH23 and GH25 families include enzymes called lysozyme known to lyse the PG. GH73 family enzymes showed a similar folding as GH23 and GH102, 103 and 104 families showed similar catalytic activities. So, we supposed that the six GHs could be isofunctional. Therefore, to be able to synthesize and to degrade PG, an organism needs a minimal set of three genes, comprising one GT28 gene, one GT51 gene and at least one gene of the five GH families mentioned above.

To circumvent the limitations associated with the aforementioned morphological and biochemical approaches to assess the presence of PG in living organisms, we aimed to develop a post-genomic, neutral approach to depict its presence among sequenced representatives of the four domains of life [10] by screening the Carbohydrate-Active Enzymes database (CAZy) [8] for the presence of the minimal set of three genes.

## Results

**Figure 1:**
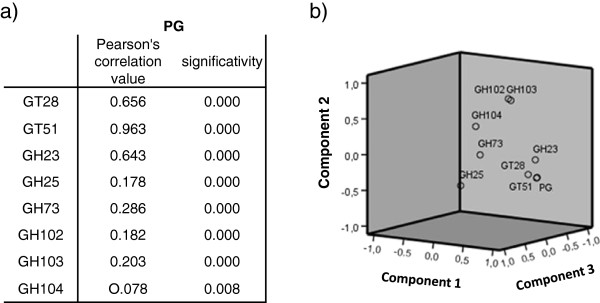
**Phylogenic 16S rDNA gene-based tree extracted from a 1,114 sequence tree from IODA.** GT51 gene gain event is represented by an orange circle. GT51 gene loss events are presented by a red square.

Whereas none of the 103 tested *Viruses* and none of the 101 tested *Archaea* genomes exhibited the 3-gene set (Table 1, Additional file 1), some representatives encode one or two genes of this 3-gene set. Indeed, the *Pseudomonas* phage JG024 and *Burkholderia ambifaria* phage Bcep F1 genomes encode one GH23 gene each. For Archaea, the *Methanosaetaconcilii* GP-6 genome contained one GH73, and the *Methanothermobacter marburgensis* str. Marburg, *Methanobacterium* sp. AL-21, *Methanothermus fervidus* DSM 2088 and *Methanopyrus kandleri* AV19 genomes encode one GT28 gene. Among 42 tested Eukaryota*,* only the *Micromonas* sp. genome encodes GT28, GT51 and GH103 (Table 1, Figure 1, Additional file 1). A total of 4 other photosynthetic eukaryotic genomes do not contain the complete 3-gene set but do encode a portion of these genes: the *Ostreococcus lucimarinus* CCE9901 and *Oryza sativa japonica* group nuclear genomes encode one and four GT28 genes, respectively; and the *Arabidopsis thaliana* nuclear and chloroplastic genomes encode a total of four GT28 genes. The *Paulinella chromatophora* chromatophore genome encodes one GT28 and one GT51 gene. Three non-photosynthetic Eukaryota genomes encode one GH23 gene, i.e. *Cryptococcus bacillisporus* WM276, *Cryptococcus neoformans* var. *neoformans* and *Homo sapiens*. By analyzing the presence of at least one gene of the 3-gene set in 42 Eukaryota genomes, we found that these genes were significantly more present in the photosynthetic Eukaryota genomes (5/7, 71.4%) than in the non-photosynthetic Eukaryota genomes (3/35, 8.5%) (P-value=0.0001). Comparing the presence of each gene family between Bacteria and the other domains of life yielded a significant association between Bacteria and the presence of GH23, GH73, GH102, GH103, GT28 (P-value <10^-7^) and GH104 (P-value <2.10^-5^). The 3-gene set was found in 1,260/1,398 (90.1%) bacteria, whereas 138 (9.9%) bacteria appeared to lack at least one of these three genes (Table 1; Additional file 2 and Additional file 3). A review of the literature indicated that all Bacteria possessing the 3-gene set have been previously demonstrated to have PG, resulting in a 100% positive predictive value of the 3-gene set for the presence of PG in an organism. For 30/138 (21.7%) organisms lacking the 3-gene set, PG information was lacking in the literature, whereas a literature review confirmed the absence of PG in 84/138 (60.9%) and the presence of PG in 24/138 (17.4%) organisms (Additional file 3). These data yielded a 77.8% negative predictive value of the 3-gene set for the presence of PG (Table 1). 

**Table 1:** Distribution of peptidoglycan metabolism genes among all of the domains of life and among 21 bacteria phyla

	**Bacteria phyla**	**GT28**	**GT51**	**GH23**	**GH25**	**GH73**	**GH102**	**GH103**	**GH104**	**Complete set**
*Archae* (n=101)		4 (3.9%)	0	0	1 (0.9%)	1 (1%)	0	0	0	0
*Viruses* (n=103)		0	0	2 (1.9%)	1 (0.9%)	0	0	0	0	0
Eukaryotes (n=42)		5 (11.9%)	2 (4.7%)	3 (7.1%)	5 (11.9%)	0	0	1 (2.4%)	0	1(2.4%)
*Bacteria* (n=1398)		1342 (96%)	1284 (91.8%)	1224 (87.5%)	419 (30%)	707 (51%)	467 (33%)	528 (37.7%)	95 (7%)	1260 (90.1%)
	*Actinobacteria* (n=136)	134 (99%)	135 (99%)	130 (95.6%)	77 (56.6%)	8 (6%)	0	0	0	133 (97.8%)
	*Aquificae* (n=9)	9 (100%)	9 (100%)	9 (100%)	0	3 (33%)	0	0	0	9 (100%)
	*Bacteroides-Chlorobi* (n=59)	58 (98%)	59 (100%)	53 (90%)	25 (42.4%)	40 (68%)	0	0	0	57 (98%)
	*Chlamydia* (n=27)	27 (100%)	0	0	0	0	0	0	0	0
	*Chloroflexi* (n=14)	9 (64%)	9 (64%)	9 (64%)	1 (7.1%)	0	0	0	0	9 (64%)
	*Cyanobacteria* (n=42)	42 (100%)	40 (95%)	32 (76%)	2 (4.7%)	7 (17%)	19 (45%)	0	23 (55%)	32 (76%)
	*Deferribacteres* (n=3)	3 (100%)	3 (100%)	3 (100%)	0	0	0	3 (100%)	0	3 (100%)
	*Deinococcus-Thermus* (n=13)	13 (100%)	13 (100%)	10 (77%)	0	0	0	0	0	10 (77%)
	*Dictyoglomi* (n=2)	2 (100%)	2 (100%)	0	0	0	0	0	0	0
	*Elusimicrobia* (n=2)	2 (50%)	2 (100%)	1 (50%)	0	0	0	0	0	1 (50%)
	*Fibrobacteres-Acidobacteria* (n=7)	6 (86%)	6 (86%)	7 (100%)	0	2 (29%)	0	0	0	6 (86%)
	*Firmicutes* (n=318)	315 (99%)	314 (99%)	264 (83%)	189 (59.4%)	256 (81%)	0	0	0	309 (97.2%)
	*Fusobacteria* (n=5)	5 (100%)	5 (100%)	3 (60%)	3 (60%)	2 (40%)	0	0	0	5 (100%)
	*Nitrospirae* (n=2)	2 (100%)	2 (100%)	2 (100%)	0	0	0	0	0	2 (100%)
	*Planctomycetes* (n=6)	3 (50%)	0	0	0	0	1 (17%)	0	0	0
	*Proteobacteria* (n=673)	664 (99%)	644 (96%)	658 (98%)	121 (18%)	370 (55%)	442 (66%)	524 (78%)	72 (11%)	644 (96%)
	*Spirochaetes* (n=27)	27 (100%)	26 (96%)	26 (96%)	1 (3.7%)	11 (41%)	4 (15%)	0	0	26 (96%)
	*Synergistetes* (n=3)	3 (100%)	2 (67%)	3 (100%)	0	0	0	0	0	2 (67%)
	*Tenericutes* (n=32)	0	0	0	0	0	0	0	0	0
	*Thermotogae* (n=11)	11 (100%)	10 (91%)	10 (91%)	0	8 (73%)	0	0	0	10 (91%)
	*Verrucomicrobia* (n=4)	4 (100%)	1 (25%)	2 (50%)	0	0	0	0	0	0
	Unclassified (n=3)	3 (100%)	2 (67%)	2 (67%)	0	0	1 (33%)	1 (33%)	0	2 (67%)

The corresponding percentage of the genome explored is indicated in parentheses.

**Figure 2:**
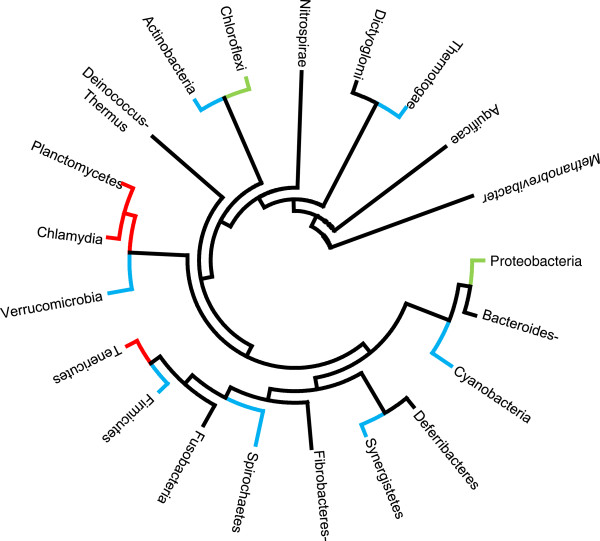
**Multiple variable analysis of peptidoglycan metabolism genes. a**) Pearson correlation test results. We compared the absence of each gene with the absence of PG. We excluded values obtained from genomes with no information for PG. **b**) Principal component analysis results. We compared the absence of each gene with the absence of PG. We excluded values obtained from genomes with no information for PG.

The Pearson correlation test indicated a significant correlation between the absence of any gene of the 3-gene set and the absence of PG, with the highest correlation value (0.963) for GT51 (P<10^-3^), as confirmed by the principal component analysis (Figure 2). 

The phylogenetic comparative analysis yielded 13 clusters (Table 2, Additional file 4). Two of the clusters aggregated the loss of PG with some PG metabolism genes: one involved PG loss and GT51 loss, with a Pagel’s score of 60, a percentage of error close to zero and five positive dates (cluster III) and another cluster involved PG loss, the loss of GT51 and GH23 genes, with a Pagel’s score of 51, a percentage of error close to zero and four positive dates (cluster IV). 

**Table 2:** Phylogenetic analysis of the gain and loss of peptidoglycan metabolism

**Clusters**	Number of dates*	**Event types**	**Genes or function**	**Pagel’s score**	**Error percentage**
I	2	Loss	GH73	27.76	≈0%
		Gain	GH25		
II	6	Loss	GH23	65.55	≈0%
		Loss	GT51		
III	5	Loss	GT51	59.95	≈0%
		Loss	PG		
IV	4	Loss	GH23	52.35	≈0%
		Loss	GT51	50.70	≈0%
		Loss	PG	51.27	≈0%
V	2	Loss	GH103	25.10	≈0%
		Loss	GH102		
VI	2	Gain	GH73	9.79	<5%
		Gain	GH25		
VII	2	Loss	GT51	1999945.66	≈0%
		Loss	GT28		
VIII	2	Loss	GH23	3.34	<50%
		Gain	GH73		
IX	2	loss	GH104	23.29	≈0%
		loss	GH25		
X	2	Gain	GH103	6.27	<20%
		Gain	GH73		
XI	2	Loss	GH25	23.44	≈0%
		Loss	GH23		
XII	2	Loss	GH102	19.18	<1%
		Gain	GH104		
XIII	2	Loss	GH103	25.51	≈0%
		Loss	GH73		

Pagel’s score was based on a chi^2^ test, with four freedom degrees and was applied to two events. Functional PG corresponds to the presence of PG in the cell wall. Date correspond to a node for which events were observed. *Detail of dates is given in the Additional file 4.

**Figure 3:**
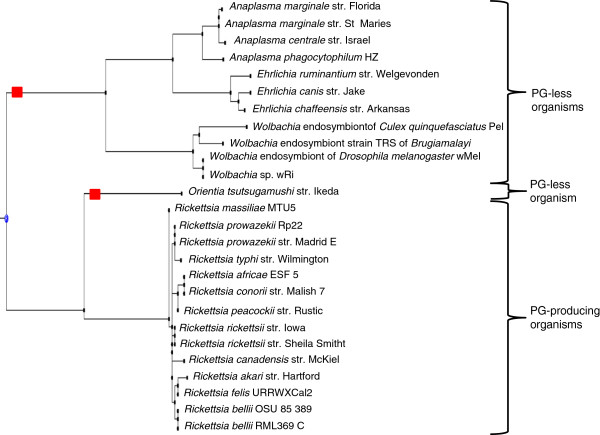
**A 16S rDNA sequence phylogenetic tree-like representation.** This representation features Bacteria phyla comprising organisms with a GT51 gene (black), phyla including some close representatives without a GT51 gene (green), phyla including isolated representatives without a GT51 gene (blue) and phyla for which all representatives lack a GT51 gene (red).

**Figure 4:**
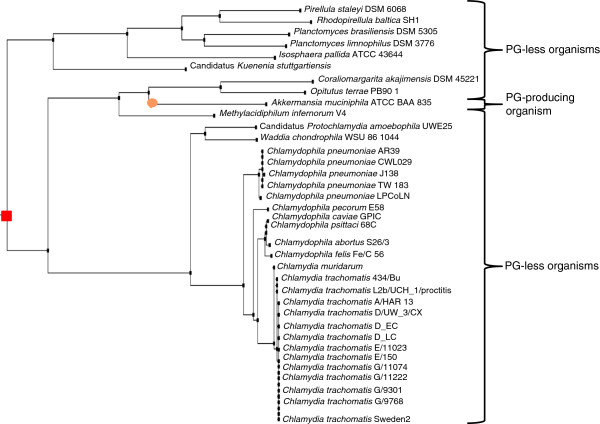
**Phylogenic 16S rDNA gene-based tree extracted from a 1,114 sequence tree from IODA.** GT51 gene loss events are presented by a red square.

Based on the GT51 criterion, 5/114 (4.4%) organisms (*Coprococcus* sp. ART55/1 [11], *Ruminococcus torques* L2-14 [11]*, Prochlorococcus marinus* str. NATL1A, *Prochlorococcus marinus* str. NATL2A [12], *Thermobaculum terrenum* ATCC BAA-798 [13] were misidentified as PG-less, lending to the absence of GT51 a 100% sensibility, a 99.53% specificity, a 94.38% positive predictive value and a 100% negative predictive value for the presence of PG in the organism. We observed that 114/1,398 (8.2%) Bacteria lacking GT51 were distributed into 13/21 (62%) *Bacteria* phyla, including *Tenericutes* (32/32; 100%), *Chlamydia* (27/27; 100%), *Planctomycetes* (6/6; 100%), *Verrucomicrobia* (3/4;75%), *Synergistetes* (1/3; 33%), *Fibrobacteres/Acidobacteria* (1/7; 14.3%), *Thermotogae* (1/11; 9%), *Chloroflexi* (5/64; 7.8%), *Cyanobacteria* (2/42; 4.8%), *Proteobacteria* (29/674; 4.3%), *Spirochaetes* (1/27; 3.7%), *Firmicutes* (4/318; 1.3%), *Actinobacteria* (1/135; 0.7%) and *Thermobaculum terrenum* (Figure 3). Among the three phyla incorporating only GT51-less bacteria, *Planctomycetes* and *Chlamydia* were closely related, and they belong to the same superphylum PVC as *Verrucomicrobia*, together comprising 75% of GT51-less organisms. The apparent absence of GT51 gene was confirmed by exploring each genome using basic local alignment search tool (BLAST) analysis [14]. The GT51 gene gain/loss events analysis indicated eight loss events and only one gain event. Among *Proteobacteria*, one loss event involved *Orientia tsutsugamusti* stc. Ikeda (PG-less organism), and the *Wolbacteria*, *Ehrlichia* and *Anaplasma* branches (Figure 4) (PG less organisms). In other phyla, loss event was observed for *Thermobaculum terrenum* ATCC BAA 798 (PG producing organism), *Prochlorococcus marinus* str. NATL1A and *Prochlorococcus marinus* str. NATL2A (PG producing organisms), *Ruminococcus torques* L2-4 (PG producing organism), the node joining of *Dehalococcoides* organisms (PG-less organisms), the node before *Ternericutes* and the node joining the *Verrucomicrobia*, *Chlamydia* and *Planctomycetes* phyla (Figure 1). The only one GT51 gene gain event was observed for *Akkermansia muciniphila* ATCC BAA 835 (Figure 1) (PG producing organism). 

The gain/loss phylogenetic trees are available on the IODA website [15].

The multivariable analysis of life style, genome size, GC content and absence or presence of PG indicated that a GC content <50%, genome size <1.5 Mb and an obligate intracellular life style were significantly correlated with the absence of PG, with odds ratios of 7.7, 80 and 19.5 and confidence intervals of 3–15.5, 42.4-152.4 and 11.7-32.5, respectively (*P*<10^-3^). Examples of such GT51-negative, PG-less obligate intracellular *Bacteria* include *Chlamydia*[16], *Anaplasma, Ehrlichia, Neorickettsia* and *Orientia*[17,18].

## Discussion

In this study, mining the CAZy database allowed the detection of a minimal set of three genes involved in PG synthesis among the four different domains of life. The fact that this complete 3-gene set was not detected in Archaea and Viruses organisms is in agreement with the previously known absence of PG in these organisms and validated our method [19]. In Archae, family GT28 genes are only very distantly related to the *bona fide* bacterial GTs involved in PG synthesis, and it is possible that the archaeal GT28 enzymes have a function unrelated to PG. In viruses, detecting a few genes potentially involved in the synthesis and in the degradation of PG was not surprising: such viruses were indeed bacterial phages in which GH genes could have recombined with the bacterial host genome [20,21] and could be used to break through the peptidoglycan layer to penetrate their bacterial hosts.

**Figure 5:**
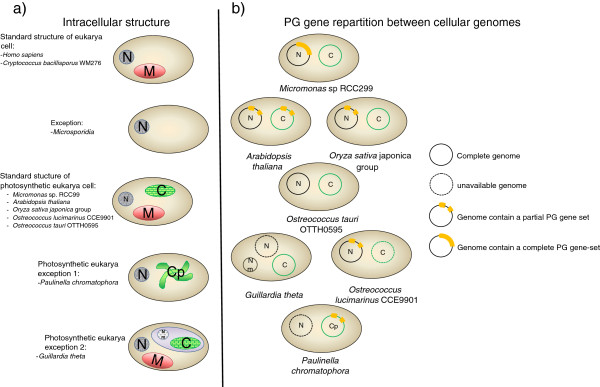
**Intracellular structure and genome distribution of the PG genes in photosynthetic Eukaryotes.** N= Nucleus, M= Mitochondria, C=Chloroplast, Cp= Chromatophore, Nm=Nucleomorph.

More surprising was the observation that the Eukaryote *Micromonas* sp. encodes a complete 3-gene set. *Micromonas* sp. is a photosynthetic picoplanktonic green alga containing chloroplasts (Figure 5) [22]. A significant association was observed between photosynthetic Eukaryotes and the presence of genes involved in PG metabolism. Chloroplasts are thought to descend from photosynthetic *Cyanobacteria* ancestors, and their presence in photosynthetic Eukaryotes is thought to result from Eukaryotes*-Cyanobacteria* symbiosis [23]. Moreover, PG has been detected in the cell wall of *Glaucophytes* chloroplasts [24,25]. We, therefore, interpreted the presence of a complete 3-gene set in *Micromonas* sp. as deriving from its chloroplast and the presence of some PG metabolism genes in other photosynthetic Eukaryotes as remnants of an ancient complete set. Additionally, the Eukaryote GT28 gene could be a remote homolog involved in plant-specific glycolipid biosynthesis and not PG metabolism. In this scenario, Eukaryotes ancestors did not encode genes for PG biosynthesis, some photosynthetic Eukaryotes further acquired such a capacity after Eukaryotes*-Cyanobacteria* symbiosis 1.5-1.2 billion years ago (Keeling 2004), and lateral genetic transfer occurred between Eukaryotes and chloroplasts [25-27]. GH23 is also encoded by free non-photosynthetic Eukaryotes; in Eukaryotes, GH23 could act as antimicrobial molecule [28]. Accordingly, we found that the minimal 3-gene set was specific for Bacteria, with a 100% positive predictive value for the presence of PG. Its predictive negative value was low, but we further determined that a lack of GT51 in the genome had a predictive negative value of 100% for the lack of PG in an organism. Moreover, our phylogenetic comparative analysis correlated the GT51 gene history and the PG history. Indeed, we observed that among the clusters including PG losses, GT51 gene losses were involved with a good Pagel’s score (cluster III and cluster IV) (Table 2). These results show that PG function is strongly linked to the presence of the GT51 gene. Thus, the GT51 gene could be used to predict the capacity of an organism to produce PG in its cell wall. 

A lack of GT51 was found in <10% of bacterial organisms. Under a parsimony hypothesis, this observation suggests that Bacteria ancestral genomes encoded GT51 and that the lack of GT51 gene in some bacteria results from loss events. Surprisingly, such loss events are observed in almost 2/3 Bacteria phyla, indicating that several independent loss events occurred during the evolutionary history of these different Bacteria phyla. These scenarios were confirmed by the gain/loss analysis featuring a GT51-containing Bacteria ancestor and eight GT51 losses. Moreover, we noticed that GT51 loss occurred in only few strains of the same species, as observed for *Prochlorococcus marinus*. Our careful examination of genomes did not find GT51 gene fragment, validating GT51 loss events which are on-going. A loss event could be counterbalanced by GT51 acquisition, as observed in *Akkermansia muciniphila* of the *Verrucomicrobia* phylum. *A. muciniphila* is living within intestinal microbiome a large microbial community where several lateral gene transfers have been reported [29]. GT51 gain/loss is a dynamic process dependent on selection pressure due to a PG advantage/disadvantage balance.

PG supports some important functions of the bacterial cell, preserving cell integrity by withstanding turgor pressure and maintaining a defined yet flexible shape. PG also anchors other cell envelope components and intimately participates in cell growth and cell division processes [1]. Nevertheless, PG is also an Achilles’ heel for *Bacteria*, as some environmental organisms produce molecules that inhibit PG synthesis. The mold *Penicillium notatum* was shown by Alexander Fleming to produce penicillin, a PG synthesis inhibitor and the first antibiotic used to treat bacterial infections in humans [30]. Vancomycin is another PG synthesis inhibitor produced by the soil bacterium *Streptomyces orientalis*[31]. However, PG is found in the vast majority of bacteria, including bacterial organisms living in the same niches as antibiotic-producing organisms. Accordingly, we observed that the absence of PG correlates with the intracellular life style and genome reduction [32]. In addition, free-living PG-less Bacteria and Archaea organisms use various osmoadapation strategies, such as the intracellular accumulation of inorganic ions, salt-tolerant enzymes or the accumulation of selected negative or neutral organic molecules [33,34] to maintain cell shape despite the absence of PG. Archaea cell walls could also contain other polymers, such as pseudomurein, methanochondroitin, heterosaccharide and glutaminylglycan, participating in the mechanical strength of the cell wall [19].

## Conclusions

The exploration of PG in bacteria shows great heterogeneity in PG content. Genome analysis with ancestral reconstructions and phylogenetic comparative analyses offer a neutral tool to explore this heterogeneity and trace the evolutionary history of PG. These analyses also allowed the identification of genes that could be used to predict functional features.

## Methods

### Screening the CAZY database

We extracted the GH23, GH73, GH102, GH103, GH10, GT28 and GT51 gene content for each genome available in CAZy in April 2011 [7], i.e., 1 398 Bacteria genomes distributed among 21 phyla, 42 Eukaryota genomes, 101 Archae genomes and 103 Viruses genomes. This database is updating regularly GenBank finished genomes for their content in carbohydrate active enzymes, providing with their EC number, gene name and product description. We then searched for the simultaneous presence of one GT28, one GT51 and at least one GH as evidence for PG metabolism. To assess the predictive value of this minimal 3-gene set, we correlated its bioinformatic detection with biological evidence for the presence of PG. We searched biological evidence for the presence of PG by screening Pubmed [35] using ‘peptidoglycan’, ‘cell wall’, ‘life style’ and the name of the genus as keywords. We further explored the HAMAP website [36], GenBank database [37] and Genome OnLine Database GOLD [38] for additional strain and genomic information. To confirm the absence of the GT51 gene in a strain, the GT51 gene nucleotide sequence of the closest strain was extracted and compared using National Center for Biotechnology Information (NCBI) BLAST to the complete genome of the strain.

### Statistical analyses

We examined the significance of the association between each gene family and each domain of life using the chi-squared test and STATCALC from EpiInfo version 6. The data were entered into an Excel spreadsheet and were analyzed using PASW statistics 17.0 (SPSS Inc., Chicago, Illinois, USA). To assess the independent factors associated with the absence of PG, binary logistic regression was performed. The dependent variable was the absence of PG, and the independent variables were life style, GC content and genome size. The goodness of fit of the results of the regression analysis was tested using the Hosmer-Lemeshow test. A correlation analysis was performed using the Pearson correlation test to assess the interaction between the absence of PG and the absence of each PG metabolism gene in the study. Principal component analysis (PCA) was used to identify colinearity between the absence of PG and the absence of each gene. The results of the PCA are shown on a factor loading plot.

### Phylogenetic tree construction

*Bacteria* phylogenetic trees were constructed based on the 16S rRNA gene sequence. An initial phylogenetic tree containing 111 16S rRNA gene sequences representing each *Bacteria* phylum was constructed and rooted using the *Archaea Methanobrevibacter smithii* 16S rRNA gene sequence. Multiple sequence alignments were performed using MUSCLE [39]. Phylogeny reconstruction of aligned sequences was performed in MEGA 5 using the neighbor-joining method and the bootstrapping method [40] after 1,000 iterations. To highlight different PG evolution events further, a second 16S rRNA gene sequence-based phylogenetic tree was constructed incorporating 1,114 sequences analyzed using the Maximum Likelihood method.

### Phylogenetic comparative analysis

The gain/loss event analysis was conducted using DAGOBAH multi-agents software system [41], integrating the PhyloPattern library [42] for Mirkin parsimony [43] ancestral node annotation and for the automatic reading of trees. The parameters were arranged to minimize the detection of gain events. To explore the existing link between the selected genes and PG, two vertical clustering calculations were conducted by DAGOBAH, one focusing on dates (framing of two speciation events) and the other focusing on feature number (gene or PG). Clusters were verified using Pagel’s method [44].

## Abbreviations

BLAST: Basic Local Alignment Search Tool; Cazy: Carbohydrate Active Enzymes; GH: Gglycoside hydrolase; GOLD: Genome OnLine Database; GT: Glycosyltransferase; HAMAP: High-quality Automated and Manual Annotation of microbial Proteomes; NCBI: National Center for Biotechnology Information; PCA: Principal component analysis; PG: Peptidoglycan.

## Competing interests

Authors have no competing interest.

## Authors’ contributions

CC, BH performed CAZY analyses. CC, PG, PP performed evolution analyses. MD designed research, critically reviewed data and drafted the manuscript. All authors contributed in writing the manuscript and reviewed and approved its final version.

## Supplementary Material

Additional file 1 Results of genomes analysis for Archaea, virus and Eukarya strains. Click here for file

Additional file 2 **Results of genomes analysis for 1398 bacteria strains.** The 1114 strains used for tree construction were highlighted in grey. PG=peptidoglycan; Set= peptidoglycan metabolism module; ND= not determined; + = presence; -= absence. Click here for file

Additional file 3 **Results of genomes analysis for 138 bacteria strains without the peptidoglycan metabolism module.** PG=peptidoglyca;. ND=not determined; += presence; -=absence. Click here for file

Additional file 4 Phylogenetic comparative analysis detailed dates. Click here for file
