# Correction: peptidoglycan: a post-genomic analysis

**DOI:** 10.1186/1471-2180-14-123

**Published:** 2014-05-27

**Authors:** Caroline Cayrou, Bernard Henrissat, Philippe Gouret, Pierre Pontarotti, Michel Drancourt

**Affiliations:** 1 Unité de Recherche sur les Maladies Infectieuses et Tropicales Emergentes, UMR CNRS 7872 IRD 198, Méditerranée Infection, Aix-Marseille-Université, Marseille, France

## Correction

After publication of [1] it has come to our attention that the figure legends associated with the figures were in the incorrect order. The corrected figure legends can be found below (see Figures 1, 2, 3, 4 and 5). In addition in the results section the sentence ‘Among 42 tested Eukaryota*,* only the *Micromonas* sp. genome encodes GT28, GT51 and GH103 (Table 1, Figure 1, Additional file 1).’ Should read ‘Among 42 tested Eukaryota*,* only the *Micromonas* sp. genome encodes GT28, GT51 and GH103 (Table 1, Figure 5, Additional file 1).’

We would like to apologize for any inconvenience.

**Figure 1:**
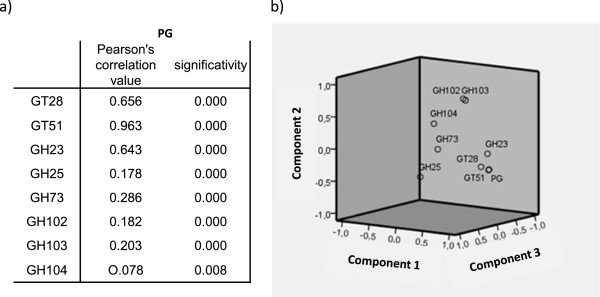
**Multiple variable analysis of peptidoglycan metabolism genes. a**) Pearson correlation test results. We compared the absence of each gene with the absence of PG. We excluded values obtained from genomes with no information for PG. **b**) Principal component analysis results. We compared the absence of each gene with the absence of PG. We excluded values obtained from genomes with no information for PG.

**Figure 2:**
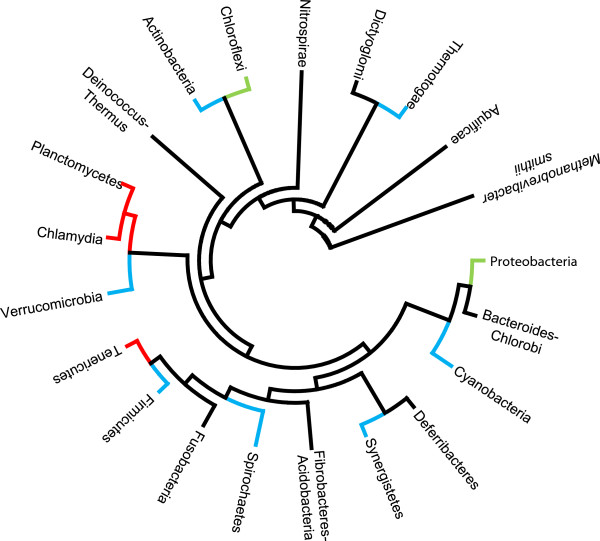
**A 16S rDNA sequence phylogenetic tree-like representation.** This representation features Bacteria phyla comprising organisms with a GT51 gene (black), phyla including some close representatives without a GT51 gene (green), phyla including isolated representatives without a GT51 gene (blue) and phyla for which all representatives lack a GT51 gene (red).

**Figure 3:**
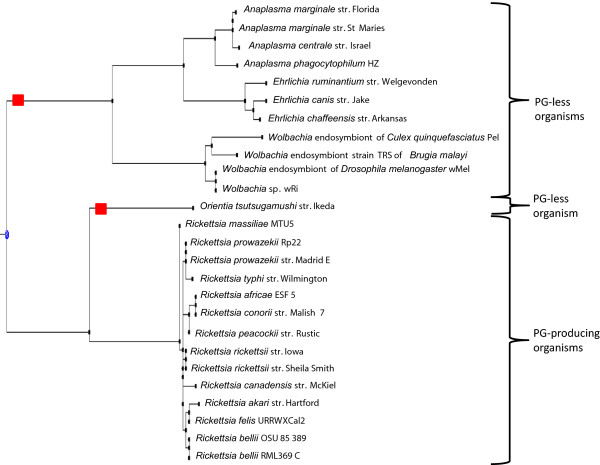
**Phylogenic 16S rDNA gene-based tree extracted from a 1,114 sequence tree from IODA.** GT51 gene loss events are presented by a red square.

**Figure 4:**
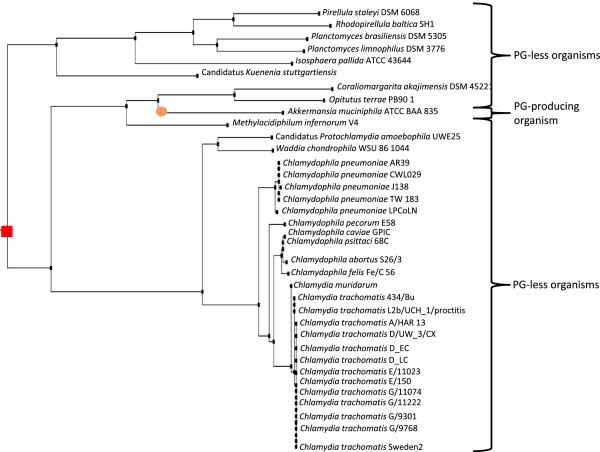
**Phylogenic 16S rDNA gene-based tree extracted from a 1,114 sequence tree from IODA.** GT51 gene gain event is represented by an orange circle. GT51 gene loss events are presented by a red square.

**Figure 5:**
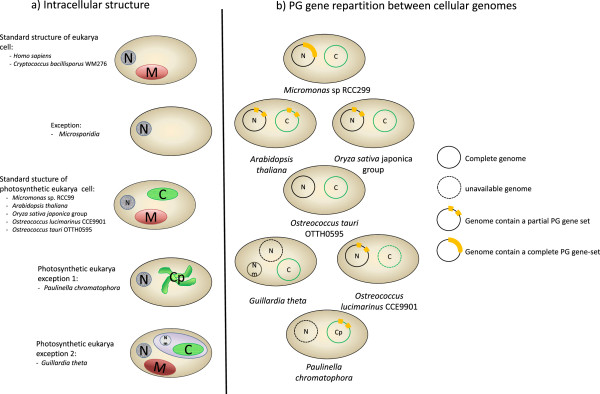
**Intracellular structure and genome distribution of the PG genes in photosynthetic Eukaryotes.** N = Nucleus, M = Mitochondria, C = Chloroplast, Cp = Chromatophore, Nm = Nucleomorph.
