# Supplementary material for: Peptidoglycan: a post-genomic analysis
Source: BMC Microbiol. 2012 Dec 18;12:294. doi: 10.1186/1471-2180-12-294 (PMC3541169; doi:10.1186/1471-2180-12-294)
Supplement: Additional file 4 — Phylogenetic comparative analysis detailed dates. [file 1471-2180-12-294-S4.docx]

**Additional file n°4.** Phylogenetic comparative analysis detailed dates.

| Clusters | Dates | Event types | Genes or function |
| --- | --- | --- | --- |
| I | Rhodospirillaceae/Azospirillum sp B510  Fusobacteriales/Leptotrichiaceae | Loss | GH73 |
|  |  | Gain | GH25 |
| II | Rickettsiales/Anaplasmataceae  Chloroflexi/Dehalococcoides McCartyI  Bacteria/*Thermobaculum terrenum* ATCC BAA 798  *Prochlorococcus marinus*/*Prochlorococcus marinus*  Bacteria/Bacteria  Bacteria/Mollicutes | Loss | GH23 |
|  |  | Loss | GT51 |
| III | Rickettsieae/*Orentia tsutsugamushi* str. Ikeda  Rickettsiales/Anaplasmataceae  Chloroflexi/Dehalococcoides McCartyI  Bacteria/Bacteria  Bacteria/Mollicutes | Loss | GT51 |
|  |  | Loss | PG |
| IV | Rickettsiales/Anaplasmataceae  Chloroflexi/Dehalococcoides McCartyI  Bacteria/Bacteria  Bacteria/Mollicutes | Loss | GH23 |
|  |  | Loss | GT51 |
|  |  | Loss | PG |
| V | Enterobacteriaceae/Candidatus *Hamiltonella defensa* 5AT *Acyrthosiphon pisum*  Gammaproteobacteria/*Wigglesworthia glossinidia* endosymbiont of *Glossina Brevipalpis* | Loss | GH103 |
|  |  | Loss | GH102 |
| VI | Alphaproteobacteria/Alphaproteobacteria  Peptococcaceae/*Syntrophobotulus glycolicus* DSM 8271 | Gain | GH73 |
|  |  | Gain | GH25 |
| VII | Chloroflexi/Dehalococcoides McCartyI  Bacteria/Bacteria | Loss | GT51 |
|  |  | Loss | GT28 |
| VIII | Coriobacteriaceae/Coriobacteriaceae  Bacteria/Bacteria | Loss | GH23 |
|  |  | Gain | GH73 |
| IX | *Escherichia coli*/ *Escherichia coli* str. K-12 substr. MG1655  Enterobacteriaceae/*Shigella dysenteriae* SD197 | loss | GH104 |
|  |  | loss | GH25 |
| X | Thermaceae/Marinithermus hydrothermalis DSM 14884  Bacteria/Bacteria | Gain | GH103 |
|  |  | Gain | GH73 |
| XI | Lactobacillales/Aerococcus urinae ACS 120-V-col10A  Lactobacillales/Carnobacterium sp 17-4 | Loss | GH25 |
|  |  | Loss | GH23 |
| XII | *Salmonella enterica* subsp. *enterica*/*Salmonella enterica* subsp. *enterica* serovar Dublin CT_02021853  Cyanobacteria/Cyanobacteria | Loss | GH102 |
|  |  | Gain | GH104 |
| XIII | Enterobacteriaceae/Candidatus *Hamiltonella defensa* 5AT *Acyrthosiphon pisum*  Marinobacter/*Marinobacter adhaerens* HP15 | Loss | GH103 |
|  |  | Loss | GH73 |

Functional PG corresponds to the presence of PG in the cell wall. Date correspond to a node for which events were observed.
